# Influence of stance width on standing balance in healthy older adults

**DOI:** 10.1007/s00415-022-11144-5

**Published:** 2022-05-09

**Authors:** Stephanie Schmidle, Alexandra Charlotte de Crignis, Manuela Stürzer, Joachim Hermsdörfer, Klaus Jahn, Carmen Krewer

**Affiliations:** 1 Human Movement Science, Department of Sport and Health Sciences, Technical University of Munich, Munich, Germany; 2 Research Department, Schön Klinik Bad Aibling, Bad Aibling, Germany

**Keywords:** Postural control, Postural balance, Older adults, Aging, Stance

## Abstract

Posturography is an objective way to systematically interpret postural control. Recent evidence suggests self-selected stance width when conducting posturography in healthy young participants, as it is easy to perform yet standardized. It is unclear, if this is similarly applicable to healthy older adults which can better serve as comparison group for persons with specific impairments, like Parkinson’s disease, who might have problems with set foot distances. The aim of this study was to investigate the influence of different stance widths on a set of parameters in healthy older adults. Twenty-four healthy elderly (65.6 ± 5.0 years, BMI 26.2 ± 4.5 kg/m^2^) participated in the study. Posturographic measurement consisted of two tests (body sway, BS; limits of stability, LoS) each assessed in five stance widths on a force platform. A series of time domain and frequency domain parameters, such as BS and LoS range, sample entropy, mean velocity, and balance functional reserve were calculated. Anthropometric parameters and self-selected stance width (mean 17.7 ± 4.7 cm) showed positive correlation. One-way repeated measures MANOVA revealed significant differences between all parameters and foot positions. Except for sample entropy in A-P dimension, univariate analysis showed significant effects of stance widths on the parameters with stronger effects on M-L dimensions. Outcomes acquired in self-selected stance width provide comparable results to standardized stance widths 20 and 30 cm. The recommendation of self-selected stance width can be adopted to older healthy subjects. Furthermore, it reflects a natural stance and includes individual body composition.

**Supplementary Information:**

The online version contains supplementary material available at 10.1007/s00415-022-11144-5.

## Introduction

An objective method of quantifying postural control is to record the trajectory of the Centre of Pressure (CoP) during standing using posturography, which indicates postural sway [1]. This can, for example, be measured as so-called *body sway test* (BS) during static unperturbed standing or during a *Limits of Stability test* (LoS) in a more dynamic condition aiming to assess the boarders of individually stabilizable standing. Deduced therefrom, different outcome parameters, like the maximal amplitude ranges of the CoP-excursions, can be calculated [1]. Suarez and colleagues [2] assessed the relationship between the sway area during LoS and BS, the *balance functional reserve* (BFR), with the intention to describe the remaining sway capacity of an individual. This parameter might facilitate the interpretation of spatial posturographic outcomes, since parameters derived from body sway alone are subject to debate about their interpretation in some cases, such as in subjects with Parkinson’s disease (PD) [3]. Moreover, numerous influential factors on posturographic outcomes such as sampling frequency, duration, repetition, visual condition, footwear, have been discussed so far. However, no consensus on stance width has been concluded yet [4, 5]. A recent publication investigating healthy young adults [6] recommends using the self-selected stance width as there was no difference in sway parameters to certain predefined positions. In addition, the authors state that it reflects a more natural and individually optimized stance taking body composition into account.

In clinical routine, posturography is used to estimate fall risks in geriatrics [1], evaluate degenerative conditions, like PD and stroke, and track progression in rehabilitation [7–9]. Since the prevalence of these pathologies increases with age [10], the first step should be therefore to determine whether this recommendation of self-selected stance can be adopted for healthy older adults. Therefore, the aim of this study was to investigate the influence of different stance widths on common posturographic time domain (mean velocity, root mean square, BS and LoS range, balance functional reserve, and sample entropy) and frequency domain (total power, and median frequency) parameters in healthy older adults to derive a stance width recommendation for the execution of posturographic measurements.

## Methods

### Participants

Twenty-four healthy older adults (≥ 60 years) participated to have a fully balanced test sequence investigating four randomized stance widths (∑ = 4! = 24). The subjects did not have any self-reported musculoskeletal or neurological disorders, history of surgeries (e.g., artificial joints), fractures or ruptures on the lower extremities within the last five years prior to study participation. Additionally, participants reported having no symptoms of dizziness or impaired balance. The study conforms to the Declaration of Helsinki. Written informed consent was obtained from all participants.

### Experimental protocol

#### Clinical assessment

Initially, sensory function (nociception, proprioception, and vibration) of the lower extremities was assessed prior to posturography to control for possible influences on postural stability, as age is a factor known to decrease foot sensitivity [11, 12]. Nociception was assessed by the sharp or blunt test on the foot soles (heel, footpads, and big toe) and proprioception by moving specific joints (hip, knee, ankle, and joint of big toe) in different directions with alternating velocities. Additionally, vibration was assessed with a Rydel-Seiffer tuning fork (values 0–8) at trochanter major, caput fibulae, malleolus lateralis and medialis, and big-toe joint. The vibration threshold was set to ≥ 3.5 [13].

#### Posturography

For posturographic measurement, participants stood unshod on a piezoelectric force platform (type 9200 AA; Mars 3.0 software; Kistler Group, CH) maintaining an upright bipedal standing position with arms hanging relaxed at their sides and heels aligned at a reference line. As previous research revealed significant influence of toe-in [14] but not toe-out or parallel [15] foot position on CoP, toe-in position was avoided. For visual orientation, an achromatic target (four vertical oriented spots of 1–2 cm diameter with 7.5–9.5 cm distance) was attached at eye level to the wall with one-meter distance to the posturography device.

The posturographic measurement consisted of two tests—the BS and LoS recording—which were repeated six times in different stance width conditions. The first stance width was self-selected, followed by four pseudorandomized stance widths (feet together, 10 cm, 20 cm, and 30 cm) and completed by the repetition of the self-selected stance width to control for learning or fatigue effects. Foot positions were copied on a paper, to ensure similarity for the self-selected position and its repetition. BS was recorded at 120 Hz for 60 s and LoS at 1000 Hz. Further details on the protocol can be extracted from Krewer et al. 2018, as the protocol was structured alike [6]. Sensory function and posturography were each assessed by one examiner to avoid interrater variation.

### Data analysis

CoP-data was exported from MARS 3.0 software and all further processing was done in Matlab (The Mathworks Inc., Natrick, US). Pre-processing consisted of down-sampling to 100 Hz and low-pass filtering at 10 Hz as proposed by Schmid et al. [16] using a 10th order zero-lag butterworth filter. The *x*-coordinates of the CoP time series represent the medio-lateral (M-L) displacement and the *y*-coordinates the anterior–posterior (A-P) displacements of the CoP.

### Posturographic outcome parameters

Body sway assessment: to describe the body sway assessment, we chose different commonly used parameters derived from the CoP time domain and frequency domain. All parameters were calculated for the A-P- and M-L-CoP time series, separately. Following parameters within the time domain were calculated:

- Mean velocity (MV): mean velocity of the CoP signal in mm/s [17].
- Root mean square (RMS): root mean square of CoP time series in mm [17].
- Range (RANGE): range of CoP displacement in mm [17].
- Sample entropy (SE): the sample entropy is a measure for assessing the regularity of a signal by calculating the probability that windows with length *m* remain similar within a tolerance *r* at the next data point [18, 19]. We used input parameters *m* = 3 and *r* = − 0.2 as previously done [20].

For the calculation of the parameters derived from the frequency domain, the CoP M-L and A-P time series were detrended by subtracting the mean and using a zero-lag high-pass 4th order butterworth filter with a cut-off frequency at 0.10 Hz. Subsequently, the power spectral density (PSD) was estimated using Welch’s method with 7 segments (each 15 s) and 50% overlapping hanning-windows. The following parameters were calculated [17]:

- Total power (TP): derived as the integral of the entire PSD in mm^2^.
- Median frequency (f50): median frequency, frequency below which the 50% of the TP is present in Hz.

Limits of Stability assessment: the LoS assessment was described regarding the ranges of the CoP displacement in A-P and M-L plane.

**Fig. 1:**
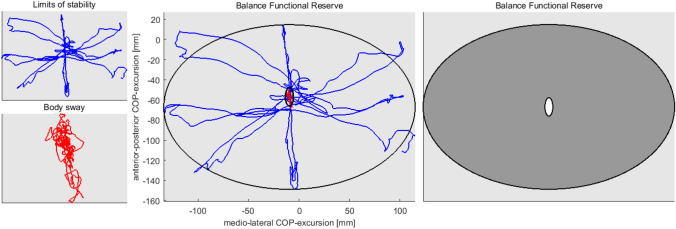
Graphical representation of the balance functional reserve. Left: CoP-trajectory during limits of stability (LoS) (top, blue) and body sway (BS) (bottom, red) at self-selected stance width; middle: LoS and BS CoP-excursion and their corresponding ellipses; right: relation of BS and LoS ellipse (share of the gray area in the total area represents the BFR). This figure represents the performance of one subject.

Combination of BS and LoS**:** derived from both assessments, the Balance Functional Reserve (BFR) was calculated. It relates the areas of the CoP-excursion during BS and LoS, calculated from the 95% prediction ellipse [21] of the BS and the LoS ellipse using the following equation ([18], see Fig. 1): $\mathrm{BFR}\left[\mathrm{\%}\right]=(1-\left(1-\left(\frac{{\mathrm{Area}}_{95\mathrm{\%BS}-\mathrm{ellipse}}}{{\mathrm{Area}}_{\mathrm{LOS}-\mathrm{ellipse}}}\right)\right)\times 100$

We additionally calculated this parameter unidimensional for the A-P and M-L plane by inserting the BS and LoS ranges instead of the ellipse area into the equation. As an example, for the A-P dimension this results in following equation:

$$\text{BFR A-P}\left[\%\right]=\left(1-\left(\frac{{\text{BS range}}_{\text{A-P}}}{{L\text{oS range}}_{\text{A-P}}}\right)\right) \times 100$$

### Statistical analysis

Pearson’s correlation was used to analyze self-selected stance width with individual’s anthropometric data. In a first step, a one-way repeated measures MANOVA (rmMANOVA) was run to test for differences between the parameters and the foot positions. In a second step, one-way rmANOVAs were calculated to determine the effect of stance width (five positions) on posturographic parameters. Greenhouse–Geisser corrected values were reported when Mauchly’s test of Sphericity was violated. Furthermore, pairwise comparisons were done using the Bonferroni confidence interval adjustment. The alpha level was set to 0.05 for all statistical analyses. Statistics were performed using SPSS (Version 26, IBM, NY, United States).

## Results

### Participants

The cohort consisted of 15 females and 9 males, mean age 65.58 ± 5.03 years (min–max 60.00–83.00), body height 1.69 ± 0.08 m (min–max 1.56–1.85), body weight 75.97 ± 18.90 kg (min–max 50.00–117.00), and BMI 26.16 ± 4.50 kg/m^2^ (min–max 18.89–35.27). Only minor / no impairments in sensory function were found: the mean vibration threshold was 6 (min–max 4–8); no impairment in nociception and proprioception was found.

### Foot positions

Analysis of foot position showed a mean self-selected stance width of 17.7 ± 4.7 cm (min–max 10–28 cm). The self-selected stance width positively correlated with participant’s body height (Pearson’s correlation coefficient *r* = 0.559, *p* = 0.002) and body weight (*r* = 0.476, *p* = 0.019). BMI showed a tendency of correlation (*r* = 0.365, *p* = 0.079).

### Influence of different foot positions on all calculated posturographic parameters

The rmMANOVA revealed a significant difference between the parameters over the five foot positions, *F*(515, 85) = 4.990, *p* = 0.000, partial *η*^2^ = 0.452. Post hoc comparison revealed that, with the exception of sample entropy in the A-P dimension, all parameters showed significant differences between foot positions.

### Influence of different foot positions on time domain parameters

#### Range: BS

RmANOVA comparing A-P ranges between all stance widths showed a significant effect of different foot positions [*F*(5, 115) = 7.288, *p* = 0.000, partial *η*^2^ = 0.241]. Sphericity was not violated, *χ*^2^ (14) = 9.058, *p* = 0.829. Comparing M-L ranges between all stance widths showed a significant effect of different foot positions [*F*(2.644, 60.818) = 92.189, *p* = 0.000, partial *η*^2^ = 0.800]. Sphericity was violated, *χ*^2^ (14) = 41.864, *p* = 0.000 (correction *ε* = 0.529). Pairwise comparisons are reported in Table 1 and visualized in Fig. 3, left).

**Table 1:**
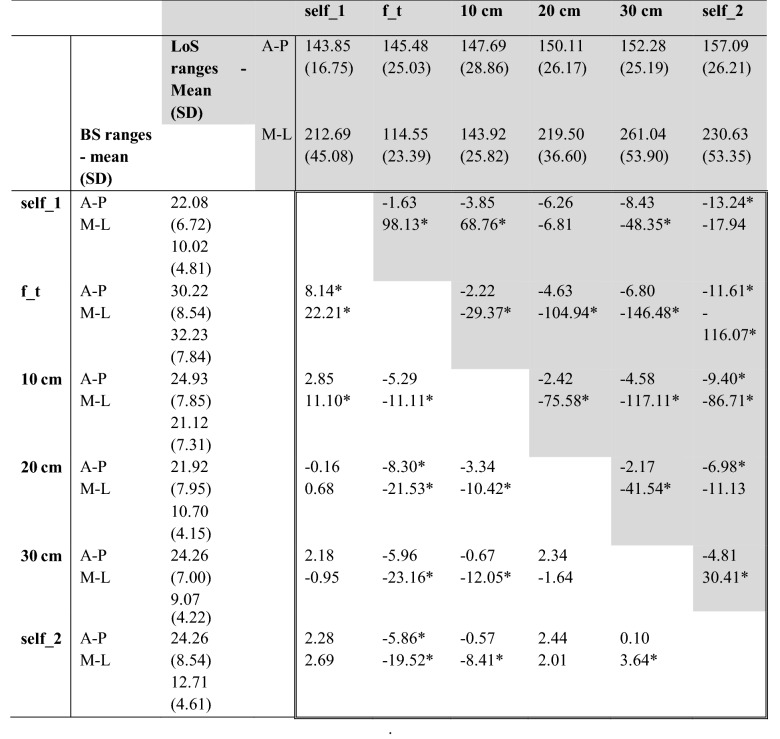
Pairwise comparison of BS and LoS ranges for each condition

Values in double framed field are mean difference (mm). Values on white background represent differences of body sway values, on gray background of LoS values

*Self_1* self-selected position, *f_t* feet together, *self_2* self-selected position repetition, *M-L* medio-lateral direction, *A-P* antero-posterior direction, *BS* body sway, *LoS* limits of stability

*Indicates significant *p*-values (*p* ≤ 0.05)

#### Range: LoS

**Fig. 2:**
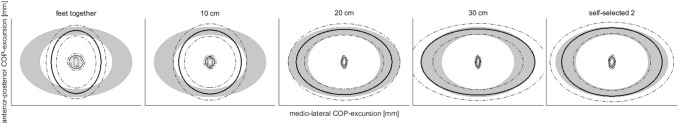
LoS and BS ellipse areas for each stance width in relation to the first self-selected position. Solid (mean) and dashed (mean ± SD) black lines present the outline of the LoS-(big) and BS-(small, in center) ellipse areas of the respective condition, gray area represents the mean ± SD ellipse area of the first self-selected position.

**Fig. 3:**
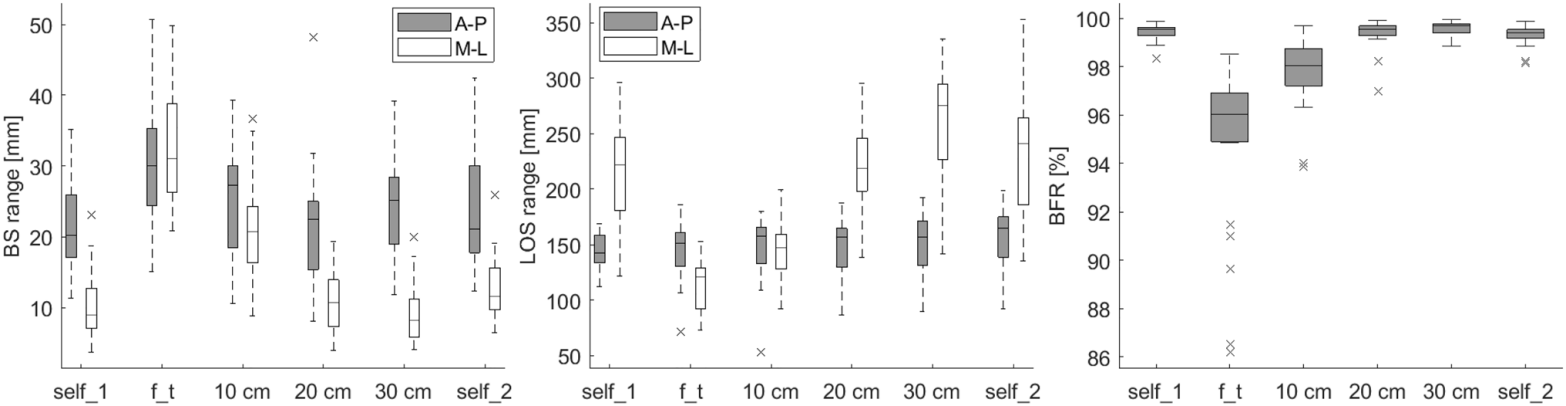
Posturographic results for each condition. *BS* body sway, *LoS* limits of stability, *BFR* balance functional reserve, *self_1* self-selected position, *f_t* feet together, *self_2* self-selected position repetition, *AP* antero-posterior direction, *MP* medio-lateral direction.

RmANOVA comparing ranges in A-P dimension between all stance widths showed a significant effect of foot position [*F*(5, 115) = 5.174, *p* = 0.000, partial *η*^2^ = 0.184]. Sphericity was not violated, *χ*^2^ (14) = 21.860, *p* = 0.083. Comparing ranges in M-L dimension between all stance widths using rmANOVA showed a significant effect of foot position [*F*(2.840, 65.313) = 128.814, *p* = 0.000, partial *η*^2^ = 0.848]. Sphericity was violated, *χ*^2^ (14) = 42.313, *p* = 0.000 (correction *ε* = 0.568). Pairwise comparisons are reported in Table 1 and visualized in Fig. 3, middle). Furthermore, the LoS and BS ellipse area for each stance width in relation to the self-selected position were calculated and are presented in Fig. 2

.

#### BFR

RmANOVA comparing the BFR between all stance widths showed significant effect of foot position [*F*(1.259, 28.965) = 45.024, *p* = 0.000, partial *η*^2^ = 0.662]. Sphericity was violated, *χ*^2^ (14) = 202.568, *p* = 0.000 (correction *ε* = 0.252). Pairwise comparisons are reported in Table 2 and visualized in Fig. 3, right). Both, BFR A-P and BFR M-L, showed significant effects of foot position (A-P *p* = 0.000, partial *η*^2^ = 0.238, *ε* = 0.643; M-L *p* = 0.000, partial *η*^2^ = 0.827, *ε* = 0.311). Pairwise comparisons are listed in Supplementary Data Table S1.

**Table 2:** Pairwise comparison of the BFR parameter for each condition

	Mean	SD	Self_1	f_t	10 cm	20 cm	30 cm	Self_2
Self_1	99.44	0.36		4.55*	1.68*	0.06	− 0.17	0.12
f_t	94.88	3.39	− 4.55*		− 2.87*	− 4.49*	− 4.72*	− 4.43*
10 cm	97.75	1.49	− 1.68*	2.87*		− 1.62*	− 1.85*	− 1.56*
20 cm	99.37	0.61	− 0.06	4.49*	1.62*		− 0.23	0.05
30 cm	99.61	0.25	0.17	4.72*	1.85*	0.23		0.29*
Self_2	99.32	0.42	− 0.12	4.43*	1.56*	− 0.05	− 0.29	

Values are mean difference (%)

*Self_1* self-selected position, *f_t* feet together, *self_2* self-selected position repetition

*Indicates significant *p*-value (*p* ≤ 0.05)

#### Mean velocity: MV

RmANOVA comparing the A-P MV between all stance widths showed a significant effect of different foot positions [*F*(5, 115) = 18.388, *p* = 0.000, partial *η*^2^ = 0.444]. Sphericity was not violated, *χ*^2^ (14) = 22.822, *p* = 0.065. RmANOVA comparing M-L MV between all stance widths showed a significant effect of different foot positions [*F*(1.752, 40.291) = 106.162, *p* = 0.000, partial *η*^2^ = 0.822]. Sphericity was violated, *χ*^2^ (14) = 94.217, *p* = 0.000 (correction *ε* = 0.350). Pairwise comparisons are reported in Supplementary Data Table S2.

#### Root mean square: RMS

RmANOVA comparing the A-P RMS between all stance widths showed significant effect of foot position [*F*(5, 115) = 5.212, *p* = 0.000, partial *η*^2^ = 0.185]. Sphericity was not violated, *χ*^2^ (14) = 19.312, *p* = 0.156. RmANOVA comparing M-L RMS between all stance widths showed a significant effect of different foot positions [*F*(2.818, 64.806) = 82.790, *p* = 0.000, partial *η*^2^ = 0.783]. Sphericity was violated, *χ*^2^ (14) = 38.086, *p* = 0.001 (correction *ε* = 0.564). Pairwise comparisons are reported in Supplemental Data Table S2.

### Influence of different foot positions on frequency domain parameters

#### Total power: TP

RmANOVA comparing total power in A-P dimension between all stance widths showed a significant effect of different foot positions [*F*(5, 115) = 8.777, *p* = 0.000, partial *η*^2^ = 0.276]. Sphericity was not violated, *χ*^2^ (14) = 15.182, *p* = 0.369. Comparing total power in M-L dimension between all stance widths showed a significant effect of different foot positions [*F*(1.237, 28.440) = 81.817, *p* = 0.000, partial *η*^2^ = 0.781]. Sphericity was violated, *χ*^2^ (14) = 241.056, *p* = 0.000 (correction *ε* = 0.247). Pairwise comparisons are reported in Supplementary Data Table S3.

#### Median frequency: f50

RmANOVA comparing f50 A-P between all stance widths showed a significant effect of different foot positions [*F*(3.023, 69.525) = 3.530, *p* = 0.019, partial *η*^2^ = 0.133]. Sphericity was violated, *χ*^2^ (14) = 34.577, *p* = 0.002 (correction *ε* = 0.605). Comparing f50 M-L between all stance widths showed a significant effect of different foot positions [*F*(3.392, 78.017) = 45.959, *p* = 0.000, partial *η*^2^ = 0.666]. Sphericity was violated, *χ*^2^ (14) = 36.561, *p* = 0.001 (correction *ε* = 0.678). Pairwise comparisons are reported in Supplementary Data Table S3.

#### Sample entropy: SE

RmANOVA comparing SE M-L between all stance widths showed a significant effect of different foot positions [*F*(2.441, 56.152) = 14.303, *p* = 0.000, partial *η*^2^ = 0.383]. Sphericity was violated, *χ*^2^ (14) = 50.363, *p* = 0.000 (correction *ε* = 0.488). Pairwise comparisons are reported in Supplementary Data Table S4.

### Individual level of foot position

To better visualize the foot positions at the individual level, we calculated the mean of the self-selected stance width and its repetition (self_1 and self_2) for each subject and set it as reference (100%). Furthermore, we expressed all other conditions as percentage of the reference and the group mean and error bar (95% confidence interval) was plotted (see Supplementary Data Fig. S1). Results depict that especially the conditions ‘feet together’ and ‘10 cm’ lead to deviations from the other conditions. Only for the A-P LoS results, this does not apply, and for the sample entropy where the 30 cm condition seems to represent a different situation.

## Discussion

The aim of this cohort study was to investigate the influence of different stance widths on posturographic parameters during standing balance in healthy older adults. Further objective was to clarify, whether recommendations for posturographic protocols in young adults [6] are equally applicable to a healthy older cohort. The cohort analyzed in this work showed no or only minor reductions in the sensory assessments, and thus is eligible to serve as healthy reference group.

The results showed that there was a significant difference between the seven calculated parameters over the five foot positions. Except for sample entropy in A-P, all parameters showed significant differences between the foot positions. During BS, the range decreased with increasing inner feet distance, which is in line with the literature examining healthy young subjects [5, 6, 14, 15, 23–27]. This work shows an additional significant influence of stance width on the LoS range, supporting previous findings by Krewer et al. [6] and Juras et al. [28]. Accordingly to earlier findings [6, 23, 28], our results demonstrate that stance width had a stronger effect on the parameter of the M-L (effect size: BS 0.80, LoS 0.85) than on the A-P dimension (effect size: BS 0.24, LoS 0.18). This is consistent, as changing the stance width results in changes of the lateral but not the sagittal aspect of the supporting surface.

Human upright stance is a generally unstable position characterized by balancing the center of mass high above the base of support. Maintaining balance is a complex motor skill based on neuromuscular control that includes the passive biomechanics, sensory inputs, and actively generated muscular torques [29]. Changes of the supporting surface causes several alterations in the neural control of bipedal stance as stance width has a major impact on frontal plane biomechanics [30]. An increasing stance width biomechanically changes the relation of body center of mass relative to the limits of the supporting surface and, therefore, permits larger excursions before reaching its boundaries [31], which is reflected by increased LoS parameters. In addition, it affects the physics of lower body motion as well as the interaction torques from upper body movements and surface [30, 31]. Increasing the stance width may also alter the stretch of muscles and tendons that stabilize the hip joints [23] and further results in an altered feedback from proprioceptors and intrinsic stiffness [30]. Those biomechanical changes may be linked to changes in sway and muscle activity [30]. Consequently, an increased base of support (feet wider apart) relates to reduced ankle joint mobility in the frontal plane [14] and, therefore, is argued to require lower levels of muscle activation for postural response [32]. This explains the decrease in spatial BS parameters. However, even though the length of the sagittal supporting surface was not changed, maximally decreased stance width (feet together) seemed to induce increased sagittal plane movements during BS. Thus, a possible explanation would be, that this biomechanically more instable position requires increased neuromuscular effort to maintain stability, which can be generated by increased movements and thus neuromuscular feedback (e.g., proprioception). This would not apply to the LoS trial, which explicitly requires weight shifting movements by the subjects. This could explain the limited effect of stance width on A-P excursion for LoS trials, as post hoc comparisons revealed mainly differences between the stance widths in comparison to the repetition of the self-selected foot position (self_2).

Bingham and colleagues [31], however, suggested that although wider stance width allows for greater center of mass excursion and greater torque generation due to mechanical leverage of the hip (see above), the increase in functional stability is only present when accompanied by appropriately scaled neural feedback. Thus, in case of an impaired nervous system, subjects may not take advantage of the intuitive benefits of wide stance due to increased neural delay, inappropriate contextual modulation, or increased sensorimotor noise [31]. But as the implementation of set feet distances during posturography becomes more difficult when the physical conditions decline due to aging and pathologies [4], a self-selected stance width would facilitate the measurement procedure. Furthermore, falling occurs most frequently due to incorrect weight shifting during standing and transferring [33], were the feet positioning is initiated self-selected by the individuum itself as well. Healthy people may only alter stance width in situations that are particularly threatening to equilibrium. Patients on the other hand, might adopt a ‘wide base’, partly unconsciously, as compensation even in supposedly non-threatening situations [23]. Therefore, various balance disorders (e.g., bilateral vestibulopathy and cerebellar ataxia) may compensate for their postural instability by increasing the stance width, which might cause difficulties in the comparability between patients and controls or even in the interpretation of posturographic outcomes. Therefore, the standardization strategy should still be adjusted to the specific purpose of research [4]. Hence, self-chosen stance width as standardized posturographic procedure should be investigated in different neurological conditions to evaluate if comparability holds true.

The repetition of the self-selected stance was conducted to detect possible fatigue or learning effects. Comparing the self-selected stance to its repetition, in LoS a significant difference appeared in A-P (*p* = 0.011) and a tendency for M-L (*p* = 0.076), possibly indicating a learning effect. On the contrary, BS rather seemed to be affected by fatigue as the ranges increased, although failing to reach significance (see Table 1). Those results indicate the presence of fatigue and learning effects, which emphasizes the value of randomizing the stance width conditions as done within this study design.

The self-selected stance widths showed large between-subject variability and significant correlations with body composition (height and weight). The relationship between stance width and body height (*r* = 0.559) was even more pronounced than reported by Mcllroy and colleagues [34] that found a small significant correlation (*r* = 0.25). In their results, weight and foot length did not account for additional variance in stance width beyond that accounted for by standing height alone. Likewise, the relation between stance width and BMI in our results is not as pronounced, it only gets significant after removing three outliers defined as > 1.5 times SD of the parameter BMI (*r* = 0.659, *p* = 0.001). One presented the lower borderline of ‘normal’ weight (BMI 18.9 kg/m^2^) and two reached the category of ‘grade I obesity’ (34.9 and 35.3 kg/m^2^). Concluding therefrom, the self-selected stance width may, to a certain extent, reflect an intuitive adaptation to body composition. It seems that this holds especially true for body compositions considered normal. In case the BMI exceeds normality, this might no longer be compensable by a broader stance width. Therefore, body height seems to be the most reliable predictor for stance width as it displays the skeletal physique, whereas the BMI can show larger fluctuations regardless of skeletal shape. Thus, the self-selected stance width seems to reflect an authentic foot position taking individual’s anthropometry (e.g., body height) into account [6].

Methodologically considered, BS trial duration could be discussed, as it was set to 60 s as recommended [35], although additional literature suggested longer recording intervals [4, 28]. However, each measurement—BS and LoS—was recorded in 6 different stance widths for 60 s with two 90 s breaks included [6]. This resulted in an overall measurement duration of almost 15 min without the set-up time or sensory assessment. A prolongation of each measurement is likely to exceed the participants acceptance and would further hinder transferability to clinical settings [4].

In conclusion, the recommendation of acquiring posturographic measurements in self-selected stance width [6] can be adopted to older healthy subjects. It seems to be an eligible method to assess posturographic parameters. Further on, assessing subjects with balance impairments in their self-selected stance width is not only likely to reflect the most natural and familiar common stance, but also possible compensation strategies for balance impairments. Thus, further research should address groups of persons prone to balance impairments (including subjects with more advanced age or pathologies) and furthermore analyze to which extent, for example, the functional reserve decreases over life span. Additionally, a direction specific calculation of BFR (functional reserve in anterior, posterior, lateral, medial) might be of future interest when analyzing subjects with specific impairments, like PD, as deviations of BFR in certain directions (e.g., posterior) could be detected.

## Supplementary Information

Below is the link to the electronic supplementary material.

Supplementary file1 (DOCX 560 KB)
