# Supplementary material for: Influence of stance width on standing balance in healthy older adults
Source: J Neurol. 2022 May 9;269(12):6228–36. doi: 10.1007/s00415-022-11144-5 (PMC9618500; doi:10.1007/s00415-022-11144-5)
Supplement: Supplementary file 1 — Supplementary file1 (DOCX 560 KB) [file 415_2022_11144_MOESM1_ESM.docx]

Influence of stance width on standing balance in healthy older adults

Journal of Neurology

Stephanie Schmidle ^1^*, Alexandra Charlotte de Crignis^2^, Manuela Stürzer^1^, Joachim Hermsdörfer ^1^, Klaus Jahn^2^, Carmen Krewer^1,2^

^1^ Human Movement Science, Department of Sport and Health Sciences, Technical University of Munich, Munich; Germany

^2^ Schön Klinik Bad Aibling, Research Department, Bad Aibling, Germany

* Author to whom correspondence should be addressed; E-Mail: stephanie.schmidle@tum.de

**Table S1.** Pairwise comparison of the BFR_AP und BFR_ML parameter for each condition.

|  |  | **Mean** | **SD** | **self_1** | **f_t** | **10 cm** | **20 cm** | **30 cm** | **self_2** |
| --- | --- | --- | --- | --- | --- | --- | --- | --- | --- |
| **self_1** | A-P  M-L | 84.52  95.15 | 4.71  2.24 |  | 6.28*  24.57* | 2.25  10.22* | -0.20  0.13 | 1.17  -1.36 | 0.65  1.08 |
| **f_t** | A-P  M-L | 78.24  70.58 | 9.34  10.44 | - 6.28*  -24.57* |  | -4.04*  -14.36* | -6.48*  -24.44* | -5.12  -25.93* | -5.63*  -23.50* |
| **10 cm** | A-P  M-L | 82.28  84.94 | 7.34  5.53 | -2.25  -10.22* | 4.04*  14.36* |  | -2.44  -10.08* | -1.08  -11.57* | -1.60  -9.14* |
| **20 cm** | A-P  M-L | 84.72  95.02 | 7.13  2.15 | 0.20  -0.13 | 6.48*  24.44* | 2.44  10.08* |  | 1.36  -1.49* | 0.85  0.94 |
| **30 cm** | A-P  M-L | 83.36  96.51 | 6.66  1.36 | -1.17  1.36 | 5.12  25.93* | 1.08  11.57* | -1.36  1.49* |  | -0.52  2.43* |
| **self_2** | A-P  M-L | 83.87  94.08 | 7.09  2.65 | -0.65  -1.08 | 5.63*  23.50* | 1.60  9.14* | -0.85  -0.94 | 0.52  -2.43* |  |

Values are mean difference (%). * indicates significant p-values (p ≤ .05). Self_1, self-selected position; f_t, feet together; self_2, self-selected position repetition, M-L, medio-lateral direction; A-P, antero-posterior direction; BFR, balance functional reserve.

|  |  |  |  | **self_1** | **f_t** | **10 cm** | **20 cm** | **30 cm** | **self_2** |
| --- | --- | --- | --- | --- | --- | --- | --- | --- | --- |
|  |  | **RMS - Mean (SD)** | M-L | 1.88  (1.09) | 5.71  (1.42) | 3.74  (1.32) | 1.92  (0.78) | 1.42  (0.73) | 2.20  (0.65) |
|  | **MVS - mean (SD)** |  | A-P | 4.13  (1.41) | 5.49  (1.58) | 4.79  (1.66) | 3.95  (1.36) | 4.48  (1.59) | 4.56  (1.68) |
| **self_1** | M-L  A-P | 3.64 (0.86)  6.77 (2.54) |  |  | -3.83*  -1.36* | -1.87*  -0.66 | -0.04  0.18 | 0.45  -0.35 | -0.33  -0.43 |
| **f_t** | M-L  A-P | 10.87 (2.97)  9.16 (2.81) |  | 7.23*  2.39* |  | 1.96*  0.70 | 3.79*  1.54* | 4.28*  1.02 | 3.50*  0.93 |
| **10 cm** | M-L  A-P | 6.79 (2.19)  7.33 (2.42) |  | 3.16*  0.57 | -4.07*  -1.83* |  | 1.83*  0.84* | 2.32*  0.31 | 1.54*  0.23 |
| **20 cm** | M-L  A-P | 3.84 (0.80)  7.12 (2.24) |  | 0.21  0.35 | -7.02*  -2.04* | -2.95*  -0.22 |  | 0.49*  -0.53 | -0.29  -0.61 |
| **30 cm** | M-L  A-P | 4.23 (0.84)  7.27 (2.38) |  | 0.60*  0.50 | -6.64*  -1.89* | -2.56*  -0.07 | 0.39  0.15 |  | -0.78*  -0.08 |
| **self_2** | M-L  A-P | 3.80 (0.80)  7.32 (2.63) |  | 0.17  0.55 | -7.07*  -1.84* | -2.99*  -0.02 | -0.04  0.20 | -0.43  0.05 |  |

**Table S2.** Pairwise comparison of MVS and RMS in M-L and A-P dimension for each condition.

Values in double framed field are mean difference (grey: mm; white: mm/s). * indicates significant p-values (p ≤ .05). Self_1, self-selected position; f_t, feet together; self_2, self-selected position repetition; M-L, medio-lateral dimension; A-P, anterior-posterior dimension; MVS, mean velocity sway; RMS, root mean square.

|  |  |  |  | **self_1** | **f_t** | **10 cm** | **20 cm** | **30 cm** | **self_2** |
| --- | --- | --- | --- | --- | --- | --- | --- | --- | --- |
|  |  | **TP - Mean (SD)** | A-P | 5.31 (3.39) | 7.69  (3.77) | 6.32  (3.95) | 4.98  (2.81) | 5.10 (3.32) | 5.15  (2.62) |
|  | **f50 - mean (SD)** |  | M-L | 1.08  (0.70) | 14.02  (6.54) | 6.08  (4.83) | 1.05 (0.91) | 0.70 (0.73) | 1.16  (0.80) |
| **self_1** | A-P  M-L | 0.22 (0.06)  0.41 (0.13) |  |  | -2.39*  -12.94* | -1.01  -5.00* | 0.33  0.04 | 0.21  0.39 | -0.15  -0.08 |
| **f_t** | A-P  M-L | 0.32 (0.14)  0.28 (0.06) |  | 0.09*  -0.13* |  | 1.37  7.94* | 2.71*  12.98* | 2.59*  13.32* | 2.54*  12.86* |
| **10 cm** | A-P  M-L | 0.27 (0.12)  0.29 (0.10) |  | 0.04  -0.13* | -0.05  0.01 |  | 1.35  5.03* | 1.22  5.39* | 1.17  4.92* |
| **20 cm** | A-P  M-L | 0.26 (0.09)  0.44 (0.14) |  | 0.04  0.02 | -0.06  0.16* | -0.01  -0.15* |  | -0.13  0.35* | -0.18  -0.12 |
| **30 cm** | A-P  M-L | 0.26 (0.10)  0.71 (0.17) |  | 0.03  0.29* | -0.06*  0.43* | -0.01  0.42* | -0.00  0.27* |  | -0.05  -0.46 |
| **self_2** | A-P  M-L | 0.26 (0.10)  0.38 (0.11) |  | 0.04  -0.04 | -0.06  0.10* | -0.00  0.09 | 0.00  -0.06 | 0.01  -0.33* |  |

**Table S3.** Pairwise comparison of total power and f50 in A-P and M-L dimension for each condition.

Values are mean difference (mm). * indicates significant p-values (p ≤ .05). Self_1, self-selected position; f_t, feet together; self_2, self-selected position repetition, M-L, medio-lateral direction; A-P, antero-posterior direction; f50, median frequency; TP, total power.

**Table S4.** Pairwise comparison of the SE parameter in M-L dimension for each condition.

|  | **Mean** | **SD** | **self_1** | **f_t** | **10 cm** | **20 cm** | **30 cm** | **self_2** |
| --- | --- | --- | --- | --- | --- | --- | --- | --- |
| **self_1** | 0.10 | 0.04 |  | 0.01 | 0.02 | 0.00 | -0.06* | 0.03 |
| **f_t** | 0.08 | 0.03 | -0.01 |  | 0.01 | -0.01 | -0.07* | 0.01 |
| **10 cm** | 0.08 | 0.02 | -0.02 | -0.01 |  | -0.02 | -0.08* | -0.01 |
| **20 cm** | 0.09 | 0.04 | -0.00 | 0.01 | 0.02 |  | -0.06* | 0.02 |
| **30 cm** | 0.20 | 0.08 | 0.06* | 0.07* | 0.08* | 0.06* |  | 0.09* |
| **self_2** | 0.07 | 0.02 | -0.03 | -0.01 | -0.01* | -0.02 | -0.09* |  |

Values are mean difference (%). * indicates significant p-value (p ≤ .05). Self_1, self-selected position; f_t, feet together; self_2, self-selected position repetition, M-L, medio-lateral direction; SE, sample entropy sway.

**Fig. S1** Results of the posturographic parameters on an individual level.


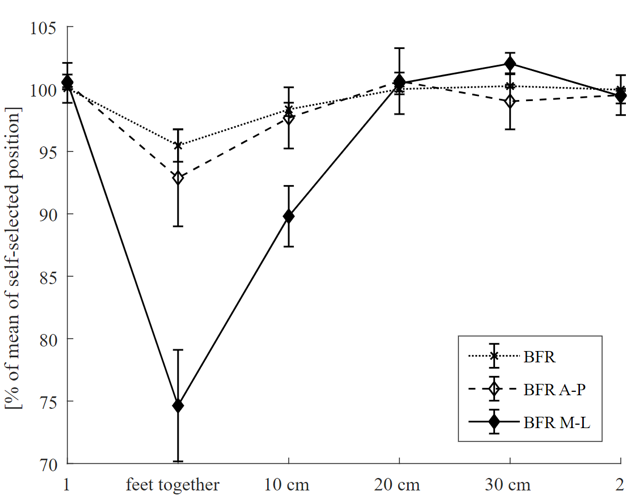

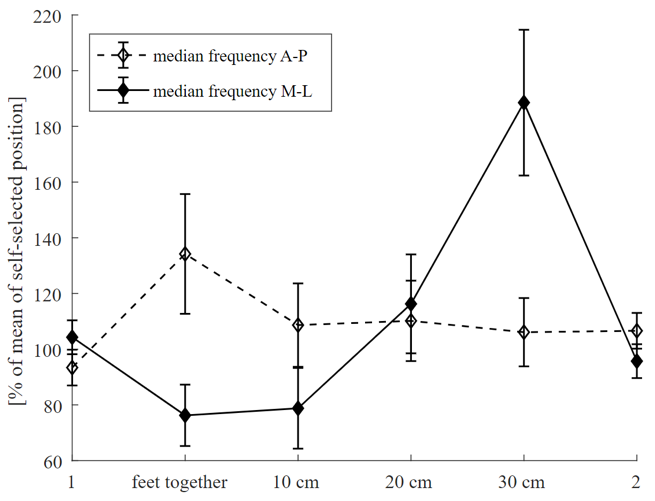


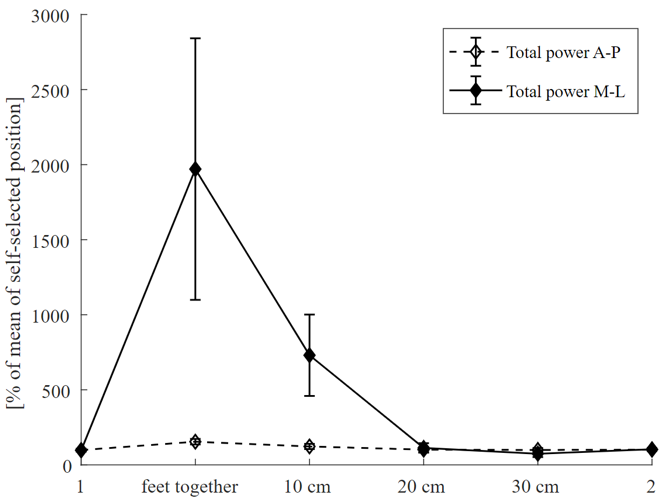

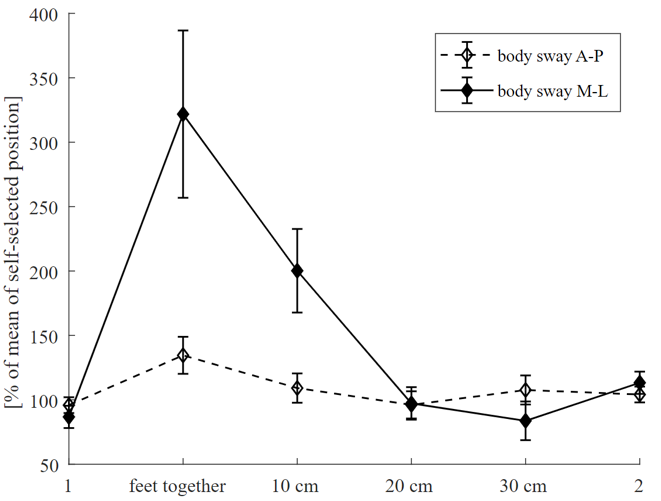

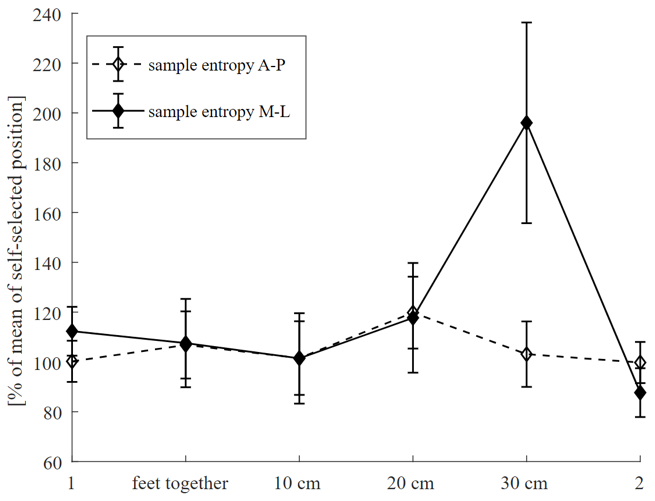

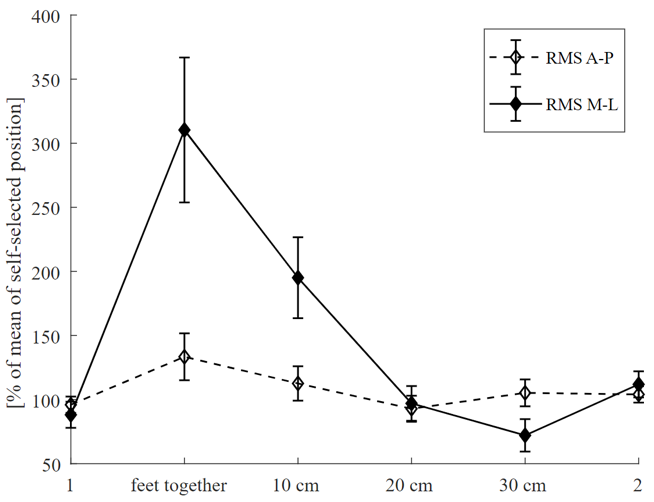

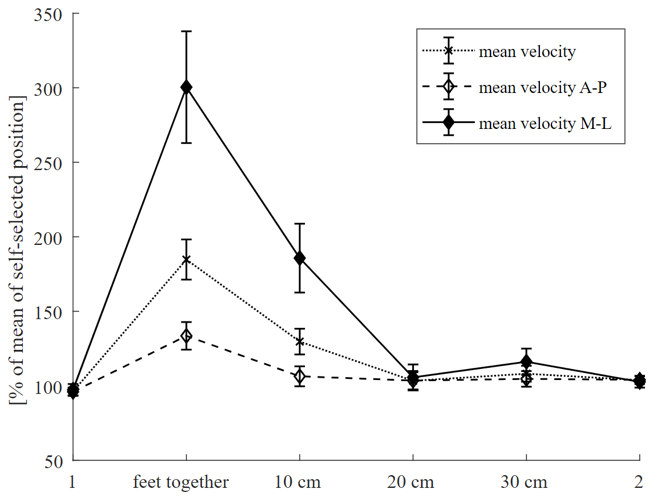

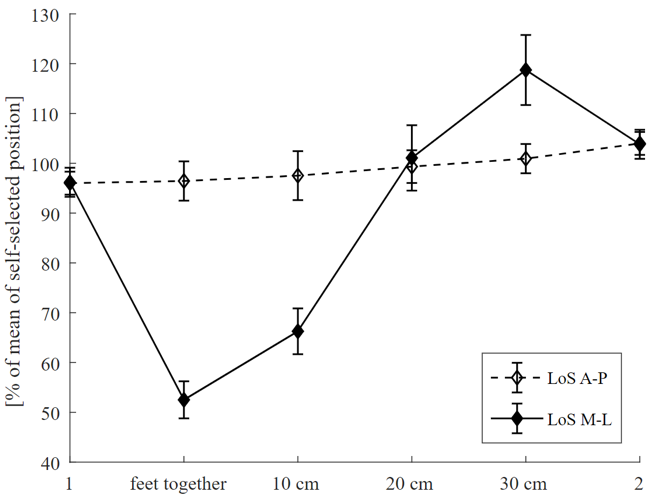


The mean of self-selected stance width and its repetition (self1, self2) was calculated for each subject and set as reference (= 100%). All conditions were then expressed as percentage of the reference and the group mean was plotted. Self1, self-selected position; self2, self-selected position repetition, M-L, medio-lateral direction; A-P, antero-posterior direction.
